# Attitudes of non-practicing chiropractors: a pilot survey concerning factors related to attrition

**DOI:** 10.1186/1746-1340-18-29

**Published:** 2010-11-04

**Authors:** Timothy A Mirtz, Jeffrey J Hebert, Lawrence H Wyatt

**Affiliations:** 1 Division of Health, Physical Education and Recreation, 414 East Clark Street--Dome 221B, University of South Dakota, Vermillion, South Dakota, 57069, USA; 2 School of Chiropractic and Sport Science, 90 South Street, Murdoch University, Murdoch, 6150, Western Australia; 3 Department of Diagnostic Imaging, Texas Chiropractic College, 5912 Spencer Highway, Pasadena, Texas 77505, USA

## Abstract

**Background:**

Research into attitudes about chiropractors who are no longer engaged in active clinical practice is non-existent. Yet non-practicing chiropractors (NPCs) represent a valid sub-group worthy of study.

**Aim:**

The purpose of this research was to assess attrition attitudes of NPCs about the chiropractic profession and develop a scale to assess such attitudes.

**Methods:**

A 48 item survey was developed using the PsychData software. This survey included 35 Likert-style items assessing various aspects of the profession namely financial, educational, psychosocial and political. An internet discussion site where NPCs may be members was accessed for recruitment purposes.

**Results:**

A total of 70 valid responses were received for analysis. A majority of respondents were male with 66% being in non-practice status for 3 to 5 years and less with 43% indicating that they had graduated since the year 2000. Most respondents were employed either in other healthcare professions and non-chiropractic education. A majority of NPCs believed that business ethics in chiropractic were questionable and that overhead expense and student loans were factors in practice success. A majority of NPCs were in associate practice at one time with many believing that associates were encouraged to prolong the care of patients and that associate salaries were not fair. Most NPCs surveyed believed that chiropractic was not a good career choice and would not recommend someone to become a chiropractor. From this survey, a 12 item scale was developed called the "chiropractor attrition attitude scale" for future research. Reliability analysis of this novel scale demonstrated a coefficient alpha of 0.90.

**Conclusion:**

The low response rate indicates that findings cannot be generalized to the NPC population. This study nonetheless demonstrates that NPCs attrition attitudes can be assessed. The lack of a central database of NPCs is a challenge to future research. Appropriate investigation of attrition within the chiropractic profession would be helpful in the analysis of attitudes regarding both chiropractic education and practice. Further research is needed in this area.

## Introduction

The chiropractic profession has frequently been promoted as a means for achieving professional success [1]. However, relatively few studies have been performed on career satisfaction or success within the chiropractic profession [2-4]. While the results of these surveys demonstrate high levels of career satisfaction among practicing chiropractors, there has yet to be any studies exploring the reasons why some chiropractors chose to leave active practice. Previous research has explored the job satisfaction of chiropractors [2-4]. Konrad et al [2] reported that global career satisfaction of chiropractors was relatively high and associated with intrinsic rewards of patient care, extrinsic rewards of compensation, and positive collegial relationships. Zhang et al [3] noted that satisfied chiropractors associated internal indicators such as practicing ethically, improving patients' health, deriving personal satisfaction, and achieving personal goals as more significant in career success than external factors represented by items such as high income. Other healthcare professions have sought to understand this dynamic [5-7]. However, these questions remain unexplored with respect to the chiropractic profession.

Non-practicing chiropractors (NPCs) represent a sub-population of the chiropractic profession, uniquely qualified to elucidate the reasons behind attrition from practice. The purpose of this study was to explore the attitudes of NPCs to understand possible reasons for their departure from active practice. The secondary purpose of this study was to develop an attrition attitude scale. Such data, it is hoped, will aid in understanding potential reasons why this group of chiropractors have left active clinical practice as a chiropractor and may increase awareness of professional issues leading to individuals leaving chiropractic practice.

## Methods

### Survey development

A 48-item survey was developed to explore NPCs attitude about the chiropractic profession. Items were developed based on four domains. It was initially determined that a NPC can be operationally defined as "a person who has a doctorate of chiropractic (DC) but is not gainfully employed in active chiropractic clinical practice." The four themes/domains were educational, financial, psychosocial and political. Each domain was determined by the authors experience in the chiropractic profession and arbitrarily chosen based on that experience. The first part of the survey consisted of demographic information (gender, age, school attended, year graduated and years out of practice). The second part asked for "yes" or "no" responses to items such as prior degree obtainment, associate experience, and if additional education was needed for a new career. Additionally, respondents were asked to specify their current career or area of employment. The third part of the survey consisted of 35 Likert-type items rated in the form of "strongly agree = 5", "moderately agree = 4", "neutral = 3", "moderately disagree = 2" and "strongly disagree = 1." These items formed the bulk of the survey from the four domains. Finally, the fourth part of the survey provided the option for respondents to include additional comments in upwards to 1000 characters with respect to their individual thoughts on or about the chiropractic profession. The survey was developed using the PsychData Online Survey program (State College, Pennsylvania).

### Survey pre-test

To help investigate the face validity of the survey, three chiropractors were asked to comment on the initial survey. Of these three chiropractors, one was retired, the other was a NPC not related to the study and another was in active practice. Each offered opinions and suggestions for modification of survey items. Minor modifications to several items were indicated for clarity with no addition or deletion of the original items.

### Sampling database

This study utilized a convenience sample of individuals with electronic mail addresses registered with an online chiropractic discussion forum known as "Chirotalk: The Skeptical Chiropractic Discussion Forum" (Chirotalk) [http://chirotalk.proboards.com/index.cgi]. At the time of sampling, the database included 2,623 members. However, 884 members were considered to be "inactive" accounts. Thus, after obtaining ethics approval from the University of South Dakota (IRB 2010.025), 1,739 individuals were contacted via electronic mail from the site administrator and invited to complete the survey. It is unknown what percentage of the 1,739 active individuals are NPCs.

### Survey delivery

This study was launched in February, 2010. Potential research participants (NPCs) were notified of the survey via electronic mail and were sent a copy of the informed consent document and an online link to the survey. Additionally, an announcement was posted on the Chirotalk website encouraging NPCs who were members of the Chirotalk forum to participate. The initial email was distributed by the website administrator with a follow-up email five days later. A third email was distributed 2 weeks after the original notification, with a final email sent 3 days later indicating that the survey was closed from further participation.

### Statistical analysis

Participant's responses via the online survey link were stored on the PsychData software site. The compiled responses were then transferred to the Statistical Package for the Social Sciences 17.0 (Chicago, Illinois) data analysis software. Descriptive statistics were thus formulated using the SPSS 17.0 program. Included in these statistical formulations was Cronbach's alpha of the items used in the survey.

## Results

### Demographic data

Initially, a total of 78 responses were received. However, eight surveys were returned void of any data leaving 70 valid surveys for analysis purposes. A valid survey was any survey that was received that was either completed or nearly completed in which to perform statistical analysis. Table 1 contains results from sections 1 and 2 of the survey. Some of the 70 surveys deemed valid did include some variables that appeared as "missing data." The results presented represent the 70 valid surveys with some of the responses having missing data as indicative from the raw numbers. Approximately 88% of the respondent NPCs were male with a majority of the sample (43%) between the ages of 31 and 40. Respondents indicated that 49% currently held an active license to practice. All of the chiropractic colleges in North America were represented, with approximately one-third of respondents receiving chiropractic degrees from Palmer College of Chiropractic or Life Chiropractic College. Additionally, five respondents listed 1 of 2 European educational programs (University of Glamorgen, and Anglo-European Chiropractic College) as the institution from which they graduated. The median year of graduation was 1995.

**Table 1:** Demographic information of non-practicing chiropractors included in this study (%/n)

Gender		Age		What school did you attend?	
Male	88% (61)	22-30	6% (4)	Life (Georgia)	17% (12)
Female	12% (8)	31-40	43% (30)	Palmer (Iowa)	16% (11)
Other*	0% (1)	41-50	31% (22)	National (Illinois)	9% (6)
		51-60	16% (11)	Western States (Oregon)	9% (6)
		61-70	4% (3)	Logan (Missouri)	7% (5)
				Other**	42% (30)

What year did you graduate from school?			How long have you been out of chiropractic?		
Median = 1995	Range (1974 to 2008)		1 to 2 years	33% (22)	
			3 to 5 years	33% (22)	
2000 to present	43% (29)		6 to 10 years	24% (16)	
1990 to 1999	28% (19)		11 to 15 years	9% (6)	
1980 to 1989	22% (15)		20 or more	1% (1)	
1970 to 1979	7% (5)		*Other	0% (3)	
Other*	0% (2)				

Did you obtain your bachelors degree before you entered chiropractic school?					
Yes			64% (44)		
No			20% (14)		
Bachelors degree during chiropractic training.			16% (11)		

Were you ever an associate in a chiropractic practice?			Were you ever a client of a practice management firm?		
Yes	65% (45)		Yes 25% (17)		
No	35% (24)		No 75% (51)		
*Other	0% (1)		*Other 0% (2)		

The current state of your chiropractic license is:					
Active	49% (33)				
Inactive	51% (34)				
*Other	0% (3)				

If you left the profession for another career, was additional schooling needed?					
Yes	48% (32)				
No	52% (35)				
*Other	0% (3)				

*Please describe your current field or profession you are now in or pursuing.					
Healthcare	30 (50%)				
Education	13 (22%)				
Other	15 (25%)				
Unemployed	2 (3%)				
*Other	10 (0%)				
*Specific career/job was initially requested and then assigned to one of the four categories.					

I had personally seen a chiropractor before entering chiropractic school.					
Yes	84% (58)				
No	16% (11)				
*Other	0% (1)				

*Missing data due to non-response of item (not included in percentage of measureable responses)

**Other schools listed not included

A total of 60 respondents of the sample specified their current field of employment. All respondents indicated that they had moved on to another career. Specifically, 48% of the respondents indicated that additional schooling was necessary to move to another career field. Four themes were generated from the data; "education", "healthcare", "other" and "unemployed."A total of 30 respondents indicated careers/jobs that were directly tied into other healthcare professions. For example, such careers/jobs included medical doctor, osteopath, nurse practitioner, physician assistant, and healthcare management. Thirteen (13) of the respondents indicated that they were employed in education in capacities ranging from high school teacher to college teaching (non-chiropractic education). Fifteen respondents listed jobs/careers that could not be categorized specifically and thus listed as "other". These included responses such as "management", "business consulting", "construction", "real estate", and "residential refuse removal." Two respondents indicated that they were currently unemployed and 2 respondents indicated that they were disabled and/or unable to work.

### Likert-scale results

Table 2 demonstrates the 35 Likert-scale items and results. When asked if "salary surveys were realistically aligned with the real world of chiropractic practice" 70% of respondents disagreed. This 70% disagreement was also found with the item of "salaries in associate practice are fair." On the issue of student loans, 64% of respondents disagreed with the statement at "student loan repayment is not a factor in career success whereas 80% agreed that "overhead expense is a contributing factor to practice success." Most respondents (64%) agreed that a "lack of benefits in a chiropractic practice" were a factor in career success." An overwhelming percentage of NPCs (83%) indicated that chiropractic is not an easy career to make money. On professional burnout, 68% of NPCs disagreed with the concept of burnout not being a factor. The title of "doctor" was an attractive feature for most NPCs (66%) as well as establishing one's own hours of work for 90% of respondents.

**Table 2:** Item specific responses from non-practicing chiropractors*

Item	Strongly agree n (%)	Moderately agree n (%)	Neutral n (%)	Moderately disagree n (%)	Strongly disagree n (%)
Financial Domain items					

Salary surveys are realistically aligned with the real world of chiropractic practice	2 (3%)	10 (14%)	9 (13%)	14 (20%)	35 (50%)

Salaries in associate practice are fair.	1 (1%)	7 (10%)	13 (19%)	21 (30%)	28 (40%)

Associates in a chiropractic practice are often encouraged to prolong the care of patients	37 (54%)	12 (17%)	8 (12%)	5 (7%)	7 (10%)

Evidence-based chiropractors can earn a fair living in chiropractic as compared to non-evidence-based chiropractors.	8 (12%)	13 (19%)	13 (19%)	15 (22%)	20 (28%)

Overhead expense is a contributing factor to practice success.	29 (42%)	26 (38%)	6 (9%)	5 (7%)	3 (4%)

A lack of benefits (paid vacation, health insurance, etc) is a factor in career success and happiness.	23 (33%)	22 (31%)	9 (13%)	6 (9%)	10 (14%)

Courses in business in the chiropractic curriculum enhance the success as a practicing chiropractor	10 (14%)	14 (20%)	13 (19%)	13 (19%)	20 (28%)

Student loan repayment is not a factor in career success in chiropractic.	4 (6%)	5 (7%)	16 (23%)	16 (23%)	29 (41%)

Chiropractic is an easy career to make money.	3 (4%)	4 (6%)	5 (7%)	11 (16%)	47 (67%)

Insurance reimbursement rates and increased paperwork are not a factor in practice success	3 (4%)	7 (10%)	8 (11%)	17 (24%)	35 (50%)

Regulations such as HIPAA and other federal laws made it difficult to maintain a thriving practice.	7 (10%)	7 (10%)	18 (26%)	21 (30%)	17 (24%)

Educational Domain items					

With a chiropractic education, one should not have trouble finding gainful employment outside of chiropractic	5 (7%)	6 (9%)	7 (10%)	15 (21%)	37 (53%)

A chiropractic education is an asset when pursuing another career.	6 (9%)	13 (19%)	7 (10%)	17 (24%)	27 (38%)

Overall, the chiropractic education has value.	12 (17%)	22 (32%)	8 (12%)	13 (19%)	14 (20%)

Chiropractic is not a good career choice.	47 (67%)	5 (7%)	6 (9%)	1 (1%)	11 (16%)

I would encourage others to become a chiropractor.	5 (7%)	4 (6%)	6 (8%)	4 (6%)	51 (73%)

Chiropractic training provides an adequate background to evaluate and assimilate the health research base	6 (9%)	19 (27%)	6 (9%)	13 (19%)	25 (36%)

There is misleading information about chiropractic before entering chiropractic school.	39 (56%)	15 (21%)	4 (6%)	4 (6%)	8 (11%)

Chiropractic schools admissions information was accurate when you entered into chiropractic school.	11 (16%)	11 (16%)	14 (20%)	10 (14%)	24 (34%)

Psychosocial Domain items					

Burn-out is not a factor in leaving active chiropractic practice.	7 (10%)	11 (16%)	4 (6%)	23 (33%)	24 (35%)

Having received care by a chiropractor is important for career success.	8 (11%)	13 (19%)	16 (23%)	11 (16%)	22 (31%)

The title of doctor is an attractive feature of being a chiropractor.	16 (23%)	30 (43%)	12 (17%)	7 (10%)	5 (7%)

Business ethics in chiropractic are perceived as questionable.	47 (67%)	9 (13%)	3 (4%)	6 (9%)	5 (7%)

There are too many chiropractors currently in practice.	40 (57%)	10 (14%)	13 (19%)	3 (4%)	4 (6%)

Setting one's own hours is an attractive feature.	40 (57%)	23 (33%)	4 (6%)	2 (3%)	1 (1%)

An associates experience in chiropractic practice is a reason for many leaving the profession.	12 (17%)	18 (26%)	19 (28%)	8 (12%)	12 (17%)

To be a good chiropractor, one must be a good adjuster.	9 (13%)	23 (33%)	11 (16%)	11 (16%)	16 (22%)

Political Domain items					

Chiropractic lacks cultural authority.	27 (40%)	23 (34%)	14 (21%)	1 (1%)	3 (4%)

If the practice of chiropractic became more like medicine's reputation I would re-enter into chiropractic practice.	14 (20%)	21 (30%)	10 (15%)	11 (16%)	13 (19%)

The political problems (non-unity, philosophy, subluxation, etc) in chiropractic are factors in being perceived as a quality clinician.	25 (36%)	15 (22%)	13 (19%)	5 (7%)	11 (16%)

The scope of practice in chiropractic is too narrow to effectively treat patients	20 (28%)	19 (27%)	6 (9%)	14 (20%)	11 (16%)

The dogma and philosophy of chiropractic are reasons to abandon active chiropractic practice.	23 (33%)	19 (27%)	8 (11%)	7 (10%)	13 (19%)

There are many chiropractors I know who are not in active practice.	16 (23%)	14 (20%)	21 (30%)	9 (13%)	10 (14%)

Chiropractors who are not in active practice are considered failures by practicing chiropractors	23 (33%)	22 (31%)	12 (17%)	6 (9%)	10 (10%)

Being injured by a chiropractor or causing injury by chiropractic treatment can be a factor in leaving active practice.	9 (13%)	19 (27%)	20 (29%)	10 (15%)	11 (16%)

*Some of the data in the columns is missing due to non-response and not included in percentage of measureable responses.

Many respondents (80%) agreed that business ethics in chiropractic were perceived as questionable and that insurance reimbursement rates as well as increased paperwork were factors towards practice success. A majority of respondents (71%) agreed that there are too many chiropractors currently in practice and 74% believed that chiropractic was a not a good career choice. Of the respondents, 79% agreed that they would not encourage others to become a chiropractor. As well, 64% of the NPCs believed that other chiropractors perceive NPCs as failures.

The question of academic value of a chiropractic degree and an individual's ability to seek employment outside the profession was explored. Only 49% agreed that overall the chiropractic education has value yet 62% disagreed with the statement that chiropractic education is an asset when pursuing another career. However, 74% of NPCs disagreed with the statement that "with a chiropractic education one should not have trouble finding gainful employment outside of chiropractic." Most respondents (77%) agreed that that there is misleading information about chiropractic before entering chiropractic school. Yet when asked if admissions information was accurate prior to when the NPC entered into chiropractic school 48% disagreed.

When NPCs were surveyed about the dogma and philosophy of chiropractic as being reasons to abandon active practice 60% of respondents agreed. Additionally, 55% of NPCs agreed that the scope of practice was too narrow to effectively treat patients. Moreover, 74% of respondents believed that chiropractic lacked cultural authority.

### Scale development

In studying the four domains mentioned (educational, political, psychosocial and financial) the attempt was made to develop attitude scales of each for possible use in future research. Each domain failed to establish an adequate coefficient alpha (internal consistency) through reliability analysis due to negative covariance. However, when all 35 items were put through reliability analysis testing 12 items emerged as internally consistent (Table 3). This 12 item scale was arbitrarily named the "chiropractor attrition attitude scale." Through reliability analysis this scale produced a coefficient alpha of 0.90.

**Table 3:** Chiropractor attrition attitude scale*

Associates in a chiropractic practice are often encouraged to prolong care of patients.
An associates experience in chiropractic practice is a reason for many leaving the profession.

Overhead expense is a contributing factor to practice success.

There is misleading information about chiropractic before entering chiropractic school.

There are too many chiropractors currently in practice.

Chiropractic is not a good career choice.

The political problems (non-unity, philosophy, subluxation, etc) in chiropractic are factors in being perceived as a quality clinician.

The dogma and philosophy of chiropractic are reasons to abandon active chiropractic practice.

Chiropractors who are not in active practice are considered failures by practicing chiropractors.

Being injured by a chiropractor or causing injury by chiropractic treatment can be a factor in leaving active practice.

Chiropractic lacks cultural authority.

A lack of benefits (paid vacation, health insurance, etc) is a factor in career success and happiness.

*Reliability analysis (coefficient alpha) of 0.90

### Written comments

In the fourth part of the survey, additional comments were provided by 48 (68.6%) respondents. Due to the heterogeneity of the comments, development of specific themes was difficult; however, several commonalities were appreciated. These included concern over student loan debt, career choice, the scientific basis of chiropractic, clinical practice, political disputes within the profession, lack of confidence in the future of the profession, inability to make an adequate living, experiences as an associate doctor, lack of acceptance by other healthcare providers, conflict with personal ethics, and the financial burden of practice along with issues of practice management.

## Discussion

The results reported by Konrad et al [2] and Zhang et al [3] present key areas of chiropractic career satisfaction. However, there also appeared to be a lack of items which could indicate potential reasons for a person to depart active practice. While some may challenge the logic of exploring the current study as a means to find "career dissatisfaction" other health professions have found value in exploring issues related to attrition and career dissatisfaction [5-7]. For example, Clem et al [8] studied career satisfaction of female emergency room physicians and included items such as "I think about leaving my current position" and "if you were to leave your current position in the next year, what would be the reason?" Additionally, Hyppola et al [9] reported that 22% of medical practitioners said that they would consider another profession if they were now beginning their university studies. Finally, Som [10] noted that 65% of attrition of graduate surgical residents was motivated by a desire to change to another specialty within medicine. Job satisfaction or surveys on chiropractic practice studies have yet to ask such questions and elucidate potential attrition or career dissatisfaction [2-4,11].

Comparisons with the results of this study and the current state of chiropractic can be made. According to the National Board of Chiropractic Examiner's (NBCE) *Job Analysis of Chiropractic *[12] nearly 60% of chiropractors have a bachelor's degree. Our study found that 64% of respondents had completed a bachelor's degree prior to chiropractic school matriculation. Our study mirrors the NBCE's data [12] with regards to gender distribution within the profession. A majority of this study's respondents graduated from Life Chiropractic College (Georgia) (17%) and Palmer College of Chiropractic (Iowa) (16%). This appears consistent with the NBCE's data [12] that reported the same two colleges (Life-GA and Palmer-IA) as the institution where most of their respondents received their degree.

The financial aspect of practice should be considered as a possible factor in attrition. The American Chiropractic Association (ACA) [13] reported that 60% of members were unsatisfied with recent actions by managed care organizations. Our study found that 74% suggested that insurance reimbursement rates were a factor in practice success. As well, the ACA reported that reduced profitability was suggested by their respondents including changes to state and federal regulations. This study however differed from the ACA as 54% of our respondents did not agree that regulations such as HIPAA (Health Insurance Portability and Accountability Act) and other federal laws made it difficult to maintain a thriving practice. As per the connection of attrition Foreman and Stahl [14] noted that any potential relationship between increasing attrition rates and financial factors is hypothetical and that further study would be needed to establish any type of a reliable association.

Rittenhouse et al [6] defined several ways other than death in which physicians attrite. These include through the means of a change in profession within clinical medicine, change to another field completely and retirement. This study identified two additional reasons for which clinicians leave active practice other than what Rittehouse et al identified [6]. These two reasons that were noted here related to either disability or professional reasons. Professional reasons were identified through the respondents written comments and included concerns over student loans, wrong career choice, the lack of a scientific basis of chiropractic, inability to identify with clinical practice, perception of low acceptance as a clinician, inability to make an adequate living, frustration over political disputes within the profession, negative associate doctor experiences, and conflict with personal ethics and practice management. A majority of respondents suggested the notion of business ethics in chiropractic as questionable. This study also found that 58% agreed that the political problems in chiropractic were factors in being perceived as a quality clinician. Such professional reasons need further examination.

Coulter et al [4] noted that chiropractors have traditionally served as a major recruiter of students for chiropractic colleges. In as much as this is an important role for an alumnus, dissatisfied chiropractors and NPCs, would tend to be unenthusiastic recruiters [4]. This study found that 74% of respondents believed that chiropractic was a not a good career choice. Of the respondents, 79% agreed that they would not encourage others to become a chiropractor. Powers [5] noted in studying the attrition of emergency medical services (EMS) professionals: "the problem is with the career itself. It is often viewed as a dead-end career or a stepping-stone to a better one in another area of medicine." This study found that 74% of NPCs disagreed with the statement that "with a chiropractic education one should not have trouble finding gainful employment outside of chiropractic." The notion that chiropractic may be viewed as a dead-end career by NPCs can be realized in that 48% of NPCs indicated that additional schooling was needed to enter another field. This study found that 62% did not believe that the chiropractic education is an asset when pursuing another career. Although 72% of respondents entered into either healthcare or education as another career, additional education would be needed to pursue some careers in these respective fields. For example, to enter medical school one would need to re-enter through the medical school's entrance requirements. Advanced standing or transfer of credits from a chiropractic education to a medical education appears to be problematic depending upon the healthcare field. In the field of education, depending upon the state one chooses to teach in, additional education and testing would be needed to qualify for a teaching certificate. One could logically conclude that chiropractors would automatically qualify for the teaching of high school biology and chemistry based largely on the basic sciences of chiropractic education. Nonetheless, many states require that certain courses need to be fulfilled as well as passing of national tests for teacher quality.

A majority of respondents (71%) agreed that there are too many chiropractors currently in practice. While some chiropractors may disagree with this sentiment there is some evidence that the chiropractic profession is in a state of oversupply. For example, in Ontario there is a long-run oversupply of practicing chiropractors [15]. The potential of oversupply of chiropractors in North America may be causing some to reevaluate their current career choice as a chiropractor. Most respondents (77%) agreed that that there is misleading information about chiropractic before entering chiropractic school yet 48% believed that admissions information was inaccurate prior to when the NPC entered into chiropractic school. One may deduce that there is a disconnect between these two findings suggestive that NPCs interpret the admissions process overall as misleading yet maybe not believing that they were misled when matriculating. However, there may be the factor of hindsight by the NPC that may need to be considered.

NPC's attitudes may have been shaped by their experiences as associate doctors. An associate is defined as a practice arrangement in which an established chiropractor most often hires a recent graduate for employment [12]. Our study differs from the NBCE [12] data as per associate practice. NBCE [12] reported that less than 1% of chiropractors practiced in an associate arrangement. This differs from our findings in that 65% of our respondents indicated that they had been an associate at some time in their career. This difference could be explained that established field practitioners were more likely to be contacted for participation by NBCE than a new graduate. The data from our study identified several items of interest as they pertain to associate practice. Seventy percent of the individuals in our survey believed that salaries in associate practice were unfair. Furthermore, 71% believed that associates in a chiropractic practice are often encouraged to prolong the care of patients. Negative experiences by those who enter into associate arrangements may be a factor in attrition.

Data found in this study could provide useful to long-range planning in the profession. As well, gaining empirical data about NPC's views could assist the profession in the quest toward cultural authority. Several authors have commented recently on the profession's lack of cultural authority [16-18]. To achieve cultural authority the chiropractic profession must be willing to internally reflect and publically report on issues related to practice attrition. Other professions have been able to internally reflect, discuss and publish findings of attrition within their own professions [5-7,9,10]. Our findings indicated that 74% of respondents believed that the chiropractic profession lacked cultural authority. However, studies of active practice chiropractors have yet to assess similar attitudes.

While the purpose of state and national associations is to represent actively practicing chiropractors, failure to acknowledge the reasons for leaving active practice could have adverse consequences. Such consequences include the inability to be cognizant of professional issues related to practice attrition. Without data to understand such issues, long-term planning may become problematic. Although the findings of this study cannot be generalized to the entire NPC population, such a study demonstrates the feasibility of undertaking a more comprehensive survey of NPCs. The development of the non-practicing chiropractor attrition attitude scale could be a starting point for the accumulation of such data. Further studies into the attitudes of NPCs should be undertaken.

## Study limitations

The current study is limited by the sampling method utilized. Given the small sample size and low rate of response, generalizations to broader populations should be made with caution. The use of a convenience sample from a single source may have biased the study results. Nonetheless, it is believed that data gathering for the sake of pilot testing was an adequate purpose. Moreover, based upon the results of this study many of the attitudes studied indicated that variations in responses did occur. Such is indicative that the items, using the Likert scale method of balancing positive and negative statements, did not produce solidly one sided responses or halo effect. Furthermore, the 0.90 alpha from the reliability analysis may be high due to the possibility of item redundancy i.e. a number of items essentially asking the same question in a slightly different way or that the scale may be too narrow in its scope to have much, if any, actual validity [19]. Furthermore, the ad hoc, a priori formulation of the statements in the Likert item section of the survey may be confounded due to biases by the authors. In addition, there is the possibility that key items of interest may have not been included that would illuminate attrition attitudes.

It is unknown if the sampling database represented only NPCs. To our knowledge, active practitioners, inactive practitioners, interested parties, other healthcare professionals, spam advertisers, students and patients could all be potential members of this database. Verification of the actual status of the anonymous respondents was not possible. It is also unknown of the national affiliation of site members. Thus, the 1,739 potential respondents cannot be claimed to be entirely representative of NPCs. This may explain the low response rate seen in this study. As well, the recruitment process may have only garnered those who still have an active interest in the chiropractic profession.

This study provides no insight into the overall incidence of attrition [10]. The incidence of attrition is best determined by data from national databases [10]. For example, data on physician attrition in the United States have been traditionally derived from the American Medical Association (AMA) Physician Masterfile or from self-reported measures of physicians' intention to retire or leave clinical practice [6]. The AMA Masterfile contains continuously updated information on all United States allopathic physicians and many osteopathic physicians, including those who are not AMA members [6]. To our knowledge none of the chiropractic associations have this type of information. It is also unknown if chiropractic college's alumni databases have information on the current status of an alumnus' active or inactive practice status as well as current employment if the alumnus is in the inactive status. Such unknowns confound the attempt to gather meaningful data through large-scale assessments on NPCs and their attitudes. It may be possible for researchers to track attrition rate by state or province [14], however, a coordinated effort by all states and provinces would need to be undertaken in order to accurately track attrition. In this instance, actual attrition could be measured and not be confounded by those chiropractors who are in transition from one state or province to another state or province.

## Conclusion

To our knowledge this is the first attempt to gather data of NPCs. NPCs, by possession of a doctor of chiropractic degree, represent a valid sub-population of chiropractors. It is concluded that NPCs, as a population, can be studied. The development of a scale to measure attrition attitudes of chiropractors should be seen as a possibility from this study. However, the lack of a central repository obtainment of such data appears to be problematic. Although this data cannot be generalized to the NPC population such data found in this study may lead to a better understanding of attrition attitudes. Future research should examine not only career satisfaction but examine career dissatisfaction and attrition attitudes.

## Conflict of interests

The authors declare that they have no competing interests.

## Authors' contributions

TAM conceptualized the idea. TAM, JJH and LHW devised items. TAM sought out colleagues for face validity. Data collection was performed by TAM. JJH and TAM performed statistical analysis. All authors contributed material. All authors approved the final manuscript.
